# Application of Multiblock Analysis on Small Metabolomic Multi-Tissue Dataset

**DOI:** 10.3390/metabo10070295

**Published:** 2020-07-17

**Authors:** Frida Torell, Tomas Skotare, Johan Trygg

**Affiliations:** 1 Computational Life Science Cluster (CLiC), Department of Chemistry, Umeå University, 90187 Umeå, Sweden; frida.torell@gmail.com (F.T.); tomas.skotare@outlook.com (T.S.); 2 Corporate Research, Sartorius, 90187 Umeå, Sweden

**Keywords:** data integration, metabolomics, multi-tissue, multiblock, joint and unique multiblock analysis (JUMBA), OnPLS, multiblock orthogonal component analysis (MOCA)

## Abstract

Data integration has been proven to provide valuable information. The information extracted using data integration in the form of multiblock analysis can pinpoint both common and unique trends in the different blocks. When working with small multiblock datasets the number of possible integration methods is drastically reduced. To investigate the application of multiblock analysis in cases where one has a few number of samples and a lack of statistical power, we studied a small metabolomic multiblock dataset containing six blocks (i.e., tissue types), only including common metabolites. We used a single model multiblock analysis method called the joint and unique multiblock analysis (JUMBA) and compared it to a commonly used method, concatenated principal component analysis (PCA). These methods were used to detect trends in the dataset and identify underlying factors responsible for metabolic variations. Using JUMBA, we were able to interpret the extracted components and link them to relevant biological properties. JUMBA shows how the observations are related to one another, the stability of these relationships, and to what extent each of the blocks contribute to the components. These results indicate that multiblock methods can be useful even with a small number of samples.

## 1. Introduction

The idea behind data integration (i.e., combining data from different sources) is that the combination of datasets is “more than the sum of its parts”, since it does not only contain the information of the respective blocks (where we by blocks mean a data matrix containing measured observations or variables from one source) but also information on their inter-relations [1]. When several data blocks are available it can be of interest to identify common variation, i.e., to integrate the data. The basis for data integration is that there is a flow of information from one block to the next. By combining the block, we obtain a more complete picture of the studied system. There are several available integration methods. The classical approach is to correlate datasets using the regression-based analysis or simple co-mapping [2]. More complex integration methods have been developed [3] such as network analysis, correlation-based analysis [4,5,6,7,8], matrix factorization methods [9,10,11,12,13,14,15,16,17], and Bayesian methods [18,19,20,21,22]. The method selection comes down to the scientific question and the types of data. The current literature includes comprehensive comparisons of several integration methods [14,22].

As mentioned, the basis for integration of data is that there is a flow of information from one block to the next. This is true when it comes to metabolites and their fluctuation between organs. In fact, metabolomics is the most dynamic omics layer [23]. In the present study, multi-tissue metabolomic samples (in this case, samples obtained by applying a metabolomics approach to six different tissue types) were integrated to find joint and unique features between and within tissue types. Data integration in this sense is about managing complexity and making it easier to interpret data from multiple sources.

Here, we present strategies for multiblock analysis (i.e., combined analysis of data from several blocks) in cases where you have a small number of samples. This can apply in case you are working with expensive samples or a pilot study. Our example dataset contains a small metabolomic multiblock dataset (six different tissue types; gut, kidney, liver, muscle, pancreas, and plasma) that has been previously studied using different approaches [24,25]. Samples were obtained from wild type mice and mice with a human point mutation in the locus of *HNF1B*, referred to as maturity onset diabetes of the young 5/renal cyst and diabetes syndrome (MODY5/RCAD). The samples were analyzed using a gas chromatography time of flight-mass spectrometry (GC TOF-MS). Throughout this study the data will collectively be referenced as blocks of data or simply blocks and each block represents one tissue type. For each mouse additional variables were also available (feces, food, glucose levels, urine, water, and weight) but were not included directly in any block, these will be referenced as metadata variables or simply metadata. They are used during the analysis and interpretation for coloring and general analysis (e.g., determining the underlying cause of the variation extracted by the models).

By combining the metabolic data from different tissue types, the overall metabolic trends were identified. For this purpose, the joint and unique multi block analysis (JUMBA) provides a flexible exploratory method that simultaneously models the globally joint, locally joint, and unique variation. The globally joint (also called simply joint or common) variation is a variation that is shared between all the analyzed blocks. The locally joint variation is a variation shared between some but not all blocks. The unique variation (sometimes called individual or distinct variation) is a variation that is only represented in a single block. OnPLS [8] (which is the model type used in the JUMBA workflow) has previously been used to integrate transcriptomic, proteomic, and metabolomic data, which displayed a multi-level oxidative stress response in the cambium of transgenic Populus plants [26]. In addition, OnPLS has been used to study the Secretory Carrier-Associated Membrane Proteins in transgenic Populus trees using transcriptomics, proteomics, GC-MS metabolomics, LC-MS metabolomics, and pyrolysis-GC/MS metabolomics [27]. The JUMBA workflow also includes interpretation tools such as visualization aids. These visualization tools reduce the complexity of the interpretation of the data integration [28]. The JUMBA workflow has been described by Surowiec et al. using a systems biology approach to study malaria [29]. In addition, JUMBA has been used for data integration as well as for calibration transfer of NIR instruments [30]. In these studies, the number of samples was higher than in the present one.

In the present study, we started by obtaining an overview of each data block separately, using single block PCA models. Thereafter, the JUMBA workflow was applied to study the globally joint and locally joint variation. Finally, we used a commonly used analysis method in the form of concatenated PCA and compared the results to the results from JUMBA. The analysis shows that the multiblock analysis can be used to provide further insights to the metabolic trends, even in cases with a low number of samples.

## 2. Results

### 2.1. Principal Component Analysis, PCA

**Figure 1:**
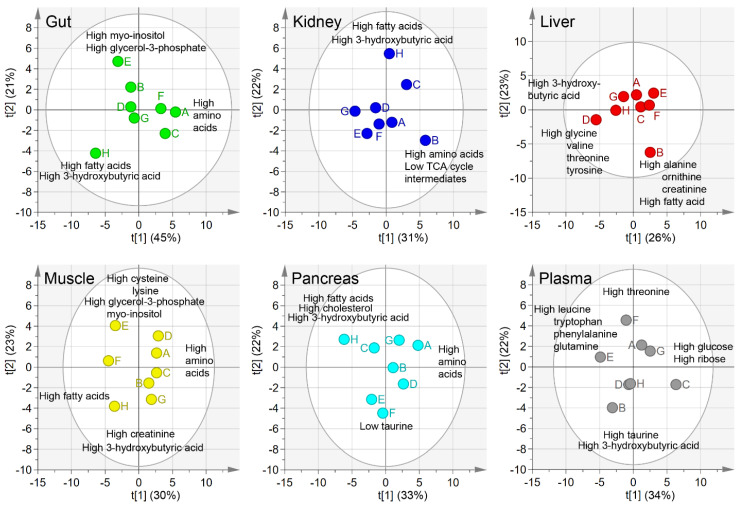
Separate principal component analysis (PCA) score plots for each tissue type. The percentages on the x- and y-axes indicated the amount of variation that was explained by each component. The underlying metabolite trends revealed by the loadings combined with the contribution plot was added as a text in the corresponding regions. Different colors were used for the tissue samples: Gut—green, kidney—blue, liver—red, muscle—yellow, pancreas—turquoise, and grey—plasma.

To obtain separate overviews of the metabolic trends in each tissue type (i.e., block), we created a two-component PCA model for each block, see Figure 1. The resulting score scatter plots showed relationships between the samples in each tissue type. The loading plots (data not shown) revealed that the metabolic patterns associated with the different sample types and contribution plots (data not shown) showed the metabolite contribution to trends observed in the score plot. By combining these sources of information, we were able to identify the metabolic trends seen in Figure 1.

More specifically, mouse H had high levels of 3-hydroxybutyric acid and fatty acids in the gut, kidney, muscle, and pancreas, as well as lower levels of amino acids in the gut and pancreas. Mouse E displayed lower levels of many metabolites, especially cholesterol and TCA cycle intermediates in the muscle, pancreas, plasma, and gut. Mouse A had high levels of amino acids in the gut, pancreas, and muscle. Mouse B was associated with high levels of amino acids in the kidney, high fatty acids in the liver, and low levels of amino acids in the plasma. Mouse D displayed intermediate levels of most metabolites in most tissues, with the exception of the high amino acid levels in the liver and muscle. Mouse F had average metabolite levels, except for higher levels of branched chain amino acids in the plasma and lower levels of myo-inositol in the gut, pancreas, and plasma. Mouse C and G displayed a more diverse metabolic pattern, showing high levels of a metabolite in some tissues and low levels in others.

### 2.2. Data Integration—JUMBA

To create a global overview of the metabolic trends, we created a single JUMBA model on all six blocks. The JUMBA model extracted the global variation (found in all tissues), locally joint variation (found in some but not all tissues), and unique variation (distinct variation found in only a single block). We used the size of the fraction of the explained variation to identify tissues with the greatest contribution to the observed trends, see Table 1. JUMBA extracted four unique components for the gut, three for the kidney, one for the liver, three for the muscle, and one for the pancreas, while no unique variation was found in the plasma. The size of the different globally joint and locally joint components is summarized in Table 1.

Due to the small number of observations, we decided to base our analysis only on the first four components, i.e., globally joint components t1 and t2, as well as locally joint components t3 and t4. Generally, we refer to the joint components in the order they appear (be they local or global), and thus the first joint component means globally joint t1, while the third joint component refers to locally joint t3, etc. To better understand the variation that was extracted by the model, we used the metadata correlation plot [28], available as Supplementary Information, Figure S1. The plot visualizes the relationship between the components and the metadata variables, and strong relationships can then be further analyzed in more detail, by inspecting the loadings and the raw data. The relationships identified using the metadata correlation plot were used for coloring JUMBA multiblock scatter plots. In general, the multiblock score scatter plot can be interpreted in the same way as a regular score scatter plot used to interpret, e.g., PCA models. However, as the underlying model is of a multiblock nature where each block contains separate scores for each component, the plot has been slightly modified.

**Figure 2:**
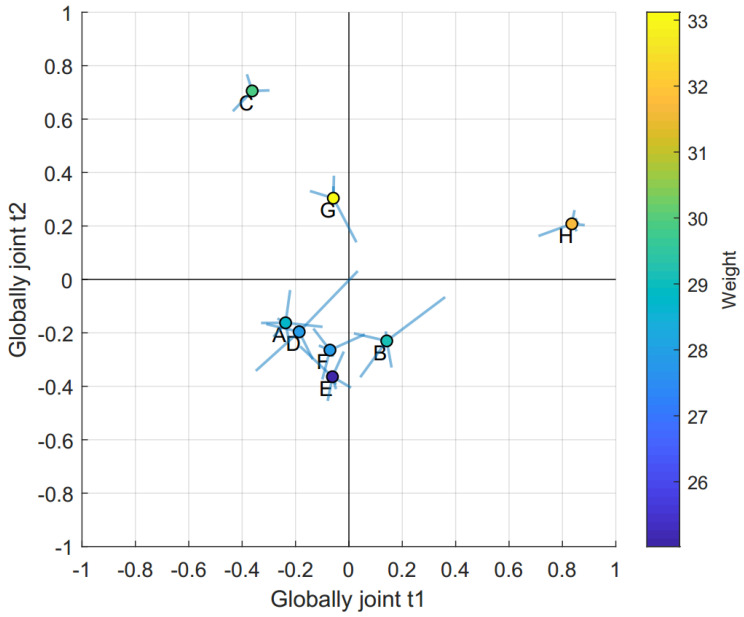
Joint and unique multiblock analysis (JUMBA) multiblock scatter plot t1 vs. t2. The multiblock scatter plot summarized the globally joint scores. The centre scatter circle represented the average score for each mouse and the whiskers represented how much each tissue type differed from this average. The colors of the scatter circles represented the weight of the animals.

As with a regular score scatter plot, each observation is represented by a symbol, commonly a circle, which may or may not be colored according to some metadata such as group belonging. From each circle, lines (called whiskers) extend to some point surrounding the symbol. The symbol itself represents the normalized mean score of the observation, while the end point of these whiskers represent the normalized score value of a single block. If a sample has long whiskers compared to other samples, it may warrant further investigation as the scores in the different blocks do not agree, maybe indicating an incorrect measurement in one or more of the blocks. Contrarily, if all whiskers are short the score does not vary much between blocks, indicating that all blocks agree. In this way the multiblock scatter plot shows the stability of samples across the blocks, while also proving the possibilities of a normal score scatter plot, e.g., the detection of trends, groupings, and outliers. Owing to a strong correlation between t2 and weight, observed in the meta data correlation plot, the multiblock scatter plot displaying globally joint t1 and t2 was colored by weight, see Figure 2.

We found that globally joint t1 did not show any strong correlation to the metadata. By inspecting the t1 score values this component was found to explain mouse H, a wild type mouse. The loadings for the corresponding components (JUMBA scores and loadings are available in Supplementary Information, Dataset and JUMBA components) revealed that mouse H was characterized by high levels of fatty acids and 3-hydroxybutyric acid and low amino acid concentrations. The trend was mainly based on the gut, pancreas, and muscles, with contributions from the other blocks, see Table 1.

The metadata correlation matrix plot revealed that globally joint t2 correlated strongly with the weight of the animals, as well as the glucose level on several of the days, see Supplementary Information, Figure S1. We found t2 to distinguish between the three heaviest mice (C, G, and H) and a cluster of the smaller mice. The model loadings revealed that the cluster of smaller mice was characterized by lower levels of fatty acids, cholesterol, ribose, and organic acids in their organs and plasma. The trend was mainly based on plasma, with contributions from all other blocks, see Table 1. We did not find any relationship between food intake and the observed trend.

**Figure 3:**
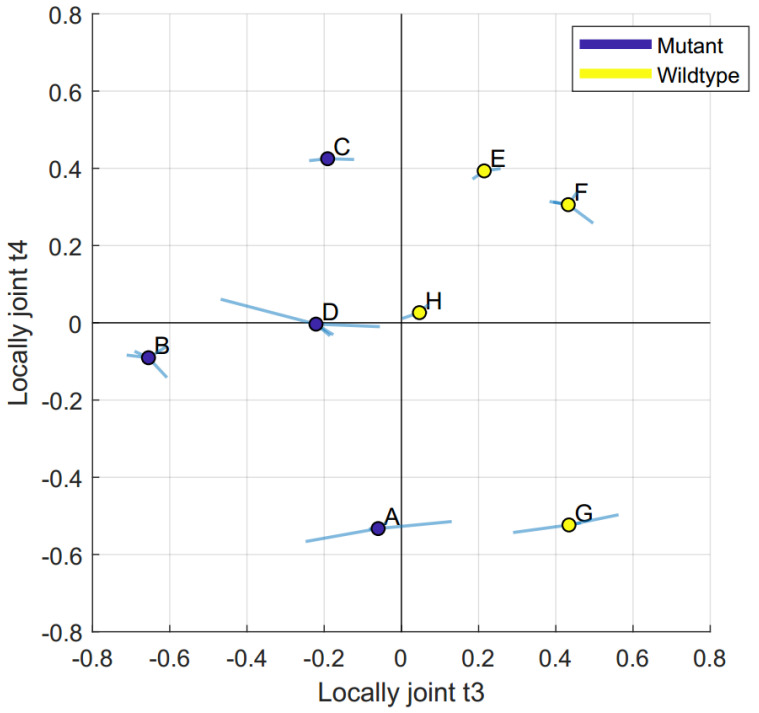
JUMBA multiblock scatter plot t3 vs. t4. The multiblock scatter plot was used to summarize locally joint components t3 and t4. The centre scatter circle represented the average score for each mouse and the whiskers represented how much each tissue type differed from this average. The circles were colored according to the genotype, showing that the locally joint t3 separates mutants from the wild type.

The metadata correlation matrix plot showed that locally joint t3 was strongly correlated to the genotype. When plotting components that did not include the same blocks (i.e., t3 vs. t4, where the kidney was only present in component t3), scores not found in both of them (i.e., kidney scores) were omitted for the creation of Figure 3. The multiblock scatter plot between locally joint t3 and t4 is colored by the genotype, see Figure 3. Locally joint t3 included all blocks except for the gut, with its main contribution coming from the kidney, see Table 1. By inspecting the JUMBA p3 loadings we found that the maturity onset diabetes of the young 5/renal cysts and diabetes syndrome (MODY5/RCAD) mice (A, B, C, and D) were characterized by higher levels of alanine, lactic acid, ornithine, tyrosine, and cholesterol in the organs (kidney, liver, muscle, and pancreas) and low levels of 3-hydroxybutyric acid, while the opposite was true for the plasma. The plasma did also have the lowest contribution to the component out of the contributing blocks.

By inspecting the JUMBA t4 scores, in combination with the p4 loadings we found the mice to be characterized by different energy states, where one group had high levels of TCA-cycle intermediates and amino acids while the other had high levels of fatty acids. Locally joint t4 included the liver, muscle, pancreas, and plasma, where the main contribution came from pancreas, see Table 1. The metadata correlation matrix plot showed that locally joint t4 had a weak correlation to weight and explained the separation between mouse C, E, and F from mouse A and G. Mouse A and G were characterized by high levels of glycine, lysine, pyroglutamic acid, taurine, tyrosine, fumaric acid, lactic acid, malic acid, myo-inositol, and 3-hydroxybutyric acid, as well as low levels of cystine, hexadecanoic acid, and palmitoleic acid.

### 2.3. Concatenated PCA

**Figure 4:**
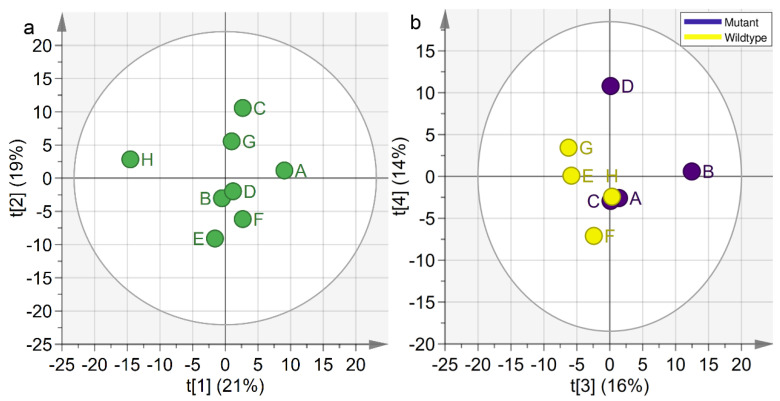
Concatenated PCA score plots. The percentages on the x- and y-axes indicated the amount of variation that was explained by each component. (**a**) Concatenated PCA t1 vs. t2. (**b**) Concatenated PCA t3 vs. t4. The circles were colored according to the genotype.

To determine whether the same trends as those seen in JUMBA could be detected using PCA, we created a PCA model on the concatenated data from all tissue types. We extracted four components to match the number of analyzed components in the JUMBA model. The score plots for t1 vs. t2 and t3 vs. t4 can be seen in Figure 4.

The first score plot (Figure 4a), depicting t1 vs. t2 provides a similar picture as JUMBA, though rotated. The first component explains the strong amino acid and fatty acid variations, observable for mouse H. The second component was similar to the weight-trend observed by JUMBA. The third and fourth concatenated PCA components (Figure 4b), did not depict strong similarities to the corresponding JUMBA components, which is not unexpected since they are not based on the same type of model procedure. For the third component, there is a trend towards separation between the genotypes, but no clear separation was observed. However, based on the p3-loading (available as Supplementary Information, Figure S2) it was visible that the gut had little impact on this component, which is similar to the third JUMBA component which included all blocks but the gut.

### 2.4. Metabolite Concentrations

To verify that the trends that were observed by the JUMBA model were truly present in the metabolomics data we studied the relative metabolite concentrations, i.e., the data the models were based on. We created dot plots showing relative concentrations in organ tissues and plasma to compare with the JUMBA model. The strongest trend was the deviation of mouse H. Metabolites where mouse H was the most extreme in three or more of the tissue types have been summarized in Supplementary Information, Figure S3. Evaluation of the relative concentrations revealed that mouse H had the highest levels of hexadecanoic acid and 3-hydroxybutyric acid and the lowest levels of alanine and serine in most of the studied organ tissues and plasma.

Another strong trend found was the size dependent separation between the mice, seen in the globally joint t2 component. Metabolites that contributed to the metabolic pattern of these three mice have been summarized in Supplementary Information, Figure S4. The three heaviest mice (C, G, and H) were mainly characterized by high levels of 3-hydroxybutyric acid, linoleic acid, stearic acid, lactic acid, ribose, and uracil. The highest levels of 3-hydroxybutyric acid were found in the organ tissues of these three mice, but they showed intermediate levels in the plasma.

We also found trends in the raw data related to the differences in relative concentrations between MODY5/RCAD mice and wild type (wt) mice (Supplementary Information, Figure S5). The plasma loadings had a negative correlation with the organ loadings. These mice were characterized by high levels of amino acids in their organ tissues and low levels in their plasma. The levels of 3-hydroxybutyric acid were found in low levels in the organ tissue samples and higher levels in the plasma samples.

## 3. Discussion

The major challenge you face when working with these low number of samples in a metabolomic study is the resulting low degrees of freedom and a lack of statistical power. Methods requiring independency of variables (metabolites are not independent), high degrees of freedom (few samples result in low degrees of freedom), or methods unable to handle the number of blocks (we had six different blocks) could not be applied. When working with a small dataset you are also at risk of overfitting the models, and cross validation may not give representative results (as excluding even a single sample is likely to have a large impact on the model) and context becomes even more important for the interpretation. For the current case, this means that a biological interpretation is required for inference.

The fact that we had samples obtained from the same mice, at the same time, analyzed for the same metabolites, provided an opportunity to integrate a small multi-tissue metabolomic data. The metabolomic data had the advantage that all the blocks contained measures of the same metabolites, which made them easier to compare. Instead of having many samples, this metabolomic dataset contained many different sources which had a stabilizing effect. By the stabilizing effect we mean that errors (e.g., run order effects or analytical artefacts) found only in a single block will have a reduced impact, as it is unlikely that the same error will randomly occur in another block. Moreover, if the same trend is observed in several of the included blocks, it adds additional credence to their relevance. While all trends should be validated against raw data and make biological sense, the fact remains that having a few samples prevents most statistical ways of validating the results (e.g., cross-validation) and this additional credence can add a degree of confidence to the results when you have a low number of samples.

Taking these limitations into consideration, we had to apply specific model creation procedures to create the JUMBA model. While the overall model procedures were the same as presented in Skotare et al. [30], there were some additional model considerations to take into account when working with this small dataset. During the JUMBA model creation, each pair of blocks is compared to extract the joint variation shared between them (as components), for an overview of the complete modelling procedure, see Supplementary Information, Figure S6.

If too many components are extracted, information not actually shared will be extracted as joint, and if too few are extracted joint variation will not be extracted. For this dataset, however, it is the case that each block is both highly similar and also has relatively few observations. As such, if too many components are extracted as joint from each pair, all variation from a block will be extracted as joint, leaving nothing as unique or residual. If all variation is extracted as joint, the model is overfitted and the joint components cannot be properly analyzed.

To avoid this, we therefore chose to simply limit the number of pairwise joint components extracted to at most one. By doing so, we expect that each pair (e.g., gut vs. plasma, gut vs. kidney, gut vs. muscle, etc.) extracts a slightly different variation from the gut, but when taken in aggregate the actual joint variation will be extracted, while still avoiding completely overfitting the model.

With this approach, we were able to extract two globally joint components and five locally joint components, indicating that this approach was a suitable setting for this particular data. We expect other combinations of pairwise joint variation to render a near identical model. Considering the noise level of the data and the number of components (as determined by PCA, we roughly expect a 50% explained variation to be extracted and we therefore set a required limit of 50% variation explained for each block), we omitted the last three joint components from further analysis, resulting in the use of four joint components (t1–t4).

The first globally joint component t1 was found to explain one of the mice. Therefore, we had to consider whether this mouse was an outlier or representing normal variation. Since we were able to determine the cause of the behavior (see further discussion below), this mouse was not considered an outlier. In this way, a modified approach to the outliers can also be required when working with this type of small dataset.

The intent of performing the multiblock analysis is to identify variations present in all or many of the blocks as well as to distinguish this variation from the variation that is unique for each block. Thereby, detecting strong trends or variations in the data, not easily found using single block investigations, e.g., using separate PCA models. As for the integration methods, we were only interested in single model methods, and therefore all hierarchical modelling methods are excluded as they consist of several separate models. We considered JIVE (joint and individual variation explained), DISCO-SCA (distinctive and common components with simultaneous-component analysis), and JUMBA (joint and unique multiblock analysis; a method based on OnPLS) [31]. From our previous research, we have found the dataset to contain the locally joint variation since the gut was not as affected by the mutation [25], therefore we were interested in methods that can handle both globally joint and locally joint, as well as unique variation. DISCO-SCA and JUMBA are both able to extract all three kinds of variations while JIVE is only able to extract the globally joint and unique variation. The method selection then came down to the choice between DISCO-SCA and JUMBA. As the authors have previously published articles using mainly JUMBA [28,29,30], and are therefore familiar with this method, it became the method of choice. Although we expect DISCO-SCA to provide similar results.

The initial PCAs provided a basis upon which it was possible to interpret some metabolic patterns of specific mice. However, the JUMBA integration method was found to be more useful in the task of gaining a combined overview of the multi-tissue dataset. A single model could be interpreted compared to six separate models, and additionally expose trends that were not as visible when interpreting the tissues separately.

More specifically, the biological relevance of the first global component t1 extracted by JUMBA was found to be the fatty acid and amino acid profile of mouse H. We hypothesized that mouse H had reduced amino acid absorption from the small intestine. Ketone bodies, such as 3-hydroxybutyric acid, are produced as part of the fatty acid metabolism when the level of carbohydrates is low which causes lipid peroxidation [32,33]. The level of carbohydrates in most tissue samples from mouse H were low, providing further evidence for our hypothesis. The process whereby fatty acids are broken down into ketone bodies is called ketogenesis and takes place in the liver [34]. For mouse H, the majority of fatty acids were found in high levels in all tissues except the liver, suggesting that the high levels of fatty acids in the remaining tissues is a means of meeting the energy demand.

Our finding that the weight of the mice influences the metabolic pattern of mice is in line with our previous research [35] where Ayala et al. found it to be ideal to compare weight-matched mice. As this is not always possible, the weight of the mice should be factored into the experimental design and the interpretation of the data. It has also been shown that body weight and composition affect metabolism [36]. An increased body weight is often caused by a higher amount of fat in the body without any increase in pure tissue mass [35]. Hence, we would expect our heavier mice to have higher fat content, which was also observed in the form of higher fatty acid and cholesterol concentrations. Therefore, we conclude that the metabolic patterns associated with higher fat concentrations should be weighed against the size of the animal.

The MODY5/RCAD mice were found to be characterized by higher levels of amino acids and fatty acids in the organs (kidney, liver, muscle, and pancreas) and lower levels of amino acids in the plasma. It has previously been shown that the gut was less affected by the mutation than the other studied tissue types [25]. This is in part due to the fact that the mutation affects a protein, hepatocyte nuclear factor 1b (hnf1b). Hnf1b is involved in the early stages of embryonic development of the kidney, liver, and pancreas [37,38,39], offering an explanation to why the genotype was explained by a locally joint, rather than one of the globally joint components.

Component t4 showed that mouse A and G are associated with higher levels of TCA cycle intermediates, amino acids, and acids that feed into the TCA cycle, as well as metabolites involved in osmoregulation and glutathione turnover [40,41]. Mouse C, E, and F were associated with higher levels of metabolites involved in lipid and fatty acids metabolism. With metabolites related to glucose and amino acid metabolism being higher in mouse A and G and metabolites related to lipid metabolism being higher in C, E, and F. Therefore, we can conclude that component four is related to the current energy metabolism of the mice.

The metabolic trends, identified by JUMBA, were also evaluated by going back to the raw data. When inspecting the raw data, it is important to remember the degree of contribution each organ has to the component, e.g., that the gut was contributing to a great extent to the first globally joint component, making the trends observed in the gut more important than the patterns seen in the plasma, for this particular component. Differences between raw data and JUMBA components are most likely related to the decomposition that is performed during model creation. Meaning that the variation that is explained by previous components is removed from the data, so that the relation between raw data and components is stronger for the first component and decreases in a descending order.

As an alternative to the multiblock approach, it is possible to perform either PCA on the concatenated variables from all the blocks or hierarchical PCA [6,42]. Both types of PCA models are expected to produce similar results to the multiblock model in cases where most variation is globally joint (i.e., include most blocks). However, the multiblock analysis provides a more detailed decomposition by allowing locally joint components to be detected. In contrast, when using concatenated PCA different variables from different organs are more likely to be summed up to determine the overall direction, meaning that the final direction of the component might not be reflected in any single block (and thus more likely to represent an actual biological trend) but rather simply be by chance. Another advantage of JUMBA is the visualization tools. In the multiblock scatter plot, larger whiskers indicate that the metabolic profiles of some samples are different and gives an indication of how well the extracted global components represent the variation observed in each block. The whiskers allow us to inspect how well the different blocks are “in agreement” for a certain observation.

In addition, JUMBA provides information regarding the block contribution to the components. Inspection of the model component overview table (see Table 1) provides information regarding which blocks share the variation and to what degree, without necessitating a detailed inspection of loadings (as in the case with the PCA variants). In this way, JUMBA allows the blocks to remain in their most explanatory form and extract variation that may be present in a subset of blocks or only in a single block.

## 4. Materials and Methods

### 4.1. Samples

Eight-month-old wild-type male mice of the mixed background 129sv x C57Bl/6N (*n* = 4) and male mice with a human point mutation in the locus of *HNF1B* (*n* = 4) (referred to as maturity onset diabetes of the young 5/renal cyst and diabetes syndrome (MODY5/RCAD)) were individually placed in metabolic cages (metabolic 5/13 cage for mice, Tecniplast^TM^, Leicester, UK) with access to food (ref. A04-10 in powder, SAFE (scientific animal food and engineering, France) and water. The animals were kept for five days in the metabolic cages: Two days as an adaptation period and three days for the experiment. On each experimental day, the animals were weighed. Food and water intake, urine volume, and the fecal weight of each mouse were recorded. On the third day of the experiment, animals were anesthetized with a ketamine and xylazine solution by an intraperitoneal injection. After 4 h of fasting, a blood sample from each animal was recovered by retro-orbital bleeding performed with a heparinised Pasteur pipette and collected in heparin tubes (Microvette CB300 LH, Sarstedt). Plasma was obtained from the blood sample by centrifugation (2000 rpm, 5 min at 20 °C), and frozen at −80 °C. The animals were killed by cervical dislocation. The gut, kidney, liver, muscle, and pancreas tissues were removed and dissected in a cold HBSS solution (Hanks’ balanced salt solution, Life Technologies, Grand Island, NY, USA). Samples of each organ were washed in HBSS, collected in cryogenic tubes, frozen in liquid nitrogen, and stored at −80 °C until analysis. Animal experiments were approved by and conducted in accordance with the French and European ethical legal guidelines and the local ethical committee for animal care (*Comité d’éthique en expérimentation animale Charles Darwin* N° 5, approval numbers N° 01508.01 and N° 04817.02).

### 4.2. Metabolic Profiling

The samples referred to as gut consisted mainly of the duodenum. The kidney, liver, and pancreas samples refer to the homogenized whole organs. The muscle samples comprised skeletal muscle tissue from the hindlimb. Detailed information about the metabolic profiling analysis, metabolite identification, and quantification can be found in the Supplementary Information, Materials and methods. The GC-MS protocol is based on two previously published studies [43,44]. To perform a more comparable data analysis we chose only to include metabolites identified in all of the tissues, thereby 35 metabolites were included in the analysis.

### 4.3. Data Analysis

To give each block an equal weight, we only used metabolites identified in both organs and plasma for the multivariate data analysis. Before any analysis we also mean-centred and scaled the data to unit variance. The methods we used for the analysis were the principal component analysis (PCA) [45,46] and JUMBA [28,29,30].

Initially, PCA was used to obtain an overview of the six different blocks, including quality control samples run in triplicates. Quality control procedures have previously been described by us [23]. For the six new PCA models the loading plots were used to reveal dominating correlation structure of the metabolic data, and the contribution plot to show the weighted difference between the mouse samples in question and the average of the model (data not shown). To study the inherent variation of the multi-tissue dataset, all blocks were concatenated, observations-wise, and analyzed using PCA, in what we referred to as concatenated PCA. For comparability four components were extracted.

OnPLS [6,7] (the model type of the JUMBA workflow [28,29,30]) is a symmetrical multiblock method. In SIMCA 16, JUMBA was implemented under the name multiblock orthogonal component analysis (MOCA). Missing values were replaced by PCA. Despite the name, in most cases including the present paper, OnPLS should be regarded as a data exploration tool, similar to PCA, not a regression method. In JUMBA, the variation is separated into areas of globally joint variation (shared by all studied datasets), locally joint variation (shared by some datasets), and areas of unique (orthogonal or distinct) variation. Variation not extracted by the model is called residuals and contains non-structured (i.e., noise) or non-linear variation. The JUMBA model provides one score and one loading vector per block included in the component. As JUMBA has highly correlated but not identical scores we visualized the joint scores using a multiblock scatter plot [28]. Each observation was represented by an average score and the whiskers showed the projection from this point onto the actual score for each block. When components that did not share the same blocks were plotted against each other, scores found in only one of the components were omitted. To create a multiblock scatter plot, the first step is to normalize the score vectors to an equal length as the magnitude of the scores can differ widely depending on the source data in each block. Thereafter, an average of the JUMBA score values of each sample is obtained, for each joint component. This average is the centre point and the protruding whiskers represent the actual normalized score value of each block, for this particular sample. The obtained values are plotted in a two-dimensional scatter plot. Further specifics on the multiblock scatter plot (and variants) can be found in Skotare et al. [28]. Model creation procedures, describing the JUMBA workflow for small data sets, are available as Supplementary information Figure S6.

### 4.4. Software

SIMCA, version 16.0 (Sartorius Stedim Data Analytics, Umeå, Sweden) was used to perform PCA. An in-house MATLAB, Version 2019a (The Mathworks, Inc., Natick, MA, USA) script was used to calculate the JUMBA model [28,29,30].

## 5. Conclusions

Our analysis demonstrates how JUMBA in combination with different visualizations give both an overview of between-mice variations as well as detailed information regarding the corresponding metabolic variations in different tissues. In addition, JUMBA provides information on block contributions to the respective components as well as how well the blocks are in agreement when it comes to the extracted components. Even when the sample count was low, JUMBA managed to identify trends within the dataset that were both biologically sound and hard to pinpoint using either separate PCA for each tissue type or a concatenated PCA.

In the current study, JUMBA managed to extract a meaningful variation which could be used to highlight concerns (e.g., the fatty acid and amino acid profile of mouse H) as well as provide meaningful characteristics (e.g., the size dependent separation as well as the genotype and differences in global energy supply). When applying the method to a dataset of low statistical power, the results should be considered as suggestions from a pilot study which could be used to steer follow-up experiments. In this way, you are able to use the smaller dataset to identify areas of interest.

Our findings support that JUMBA is suitable in the start-up of a project as a powerful tool in the familiarization process. We conclude that the properties of JUMBA, i.e., the ability to provide a single-model overview, summarizing the structure in several tissue types, while also providing confidence in the interpretation, prove especially valuable in cases with a few samples.

## Figures and Tables

**Table 1:** Model component overview. These percentages represent the amount of compartment variation that is explained by each component. For example, the first globally joint component explained 37% of the variation in the gut.

Component	Gut	Kidney	Liver	Muscle	Pancreas	Plasma
Globally joint t1	37%	14%	17%	23%	25%	12%
Globally joint t2	19%	19%	14%	18%	19%	29%
Locally joint t3		28%	17%	18%	8%	18%
Locally joint t4			11%	15%	24%	11%
Locally joint t5		19%	21%		11%	7%
Locally joint t6	25%				7%	16%
Locally joint t7			11%			7%
Residual	19%	20%	8%	26%	6%	-

## Supplementary Materials

The following are available online at https://www.mdpi.com/2218-1989/10/7/295/s1, Figure S1: Metadata correlation matrix plot; Figure S2: Concatenated PCA loading p3. Figure S3. Mouse H relative concentrations. Figure S4. Mouse C, G and H relative concentrations. Figure S5. MODY5/RCAD mutation vs. wild type (wt). Figure S6. JUMBA Workflow overview. In the Supplementary Information: Materials and methods is available. Excel document with Supplementary information dataset and JUMBA components is also available.
