# Supplementary material for: Application of Multiblock Analysis on Small Metabolomic Multi-Tissue Dataset
Source: Metabolites. 2020 Jul 17;10(7):295. doi: 10.3390/metabo10070295 (PMC7407932; doi:10.3390/metabo10070295)
Supplement: Supplementary file 1 [file metabolites-10-00295-s001.zip › Supplementary Information.docx]

Multiblock analysis on a small metabolomic multi-tissue dataset

Frida Torell^1,2,¤^, Tomas Skotare^1,¤^, Johan Trygg^1,*^

^1^ Computational Life Science Cluster (CLiC), Department of Chemistry, Umeå University, 90187, Sweden

^2^ Accelerator Lab (ACL), Karlsruhe Institute of Technology, 76344, Germany

^¤^ Equal contribution

^*^ Corresponding author: johan.trygg@umu.se

## Supplementary information


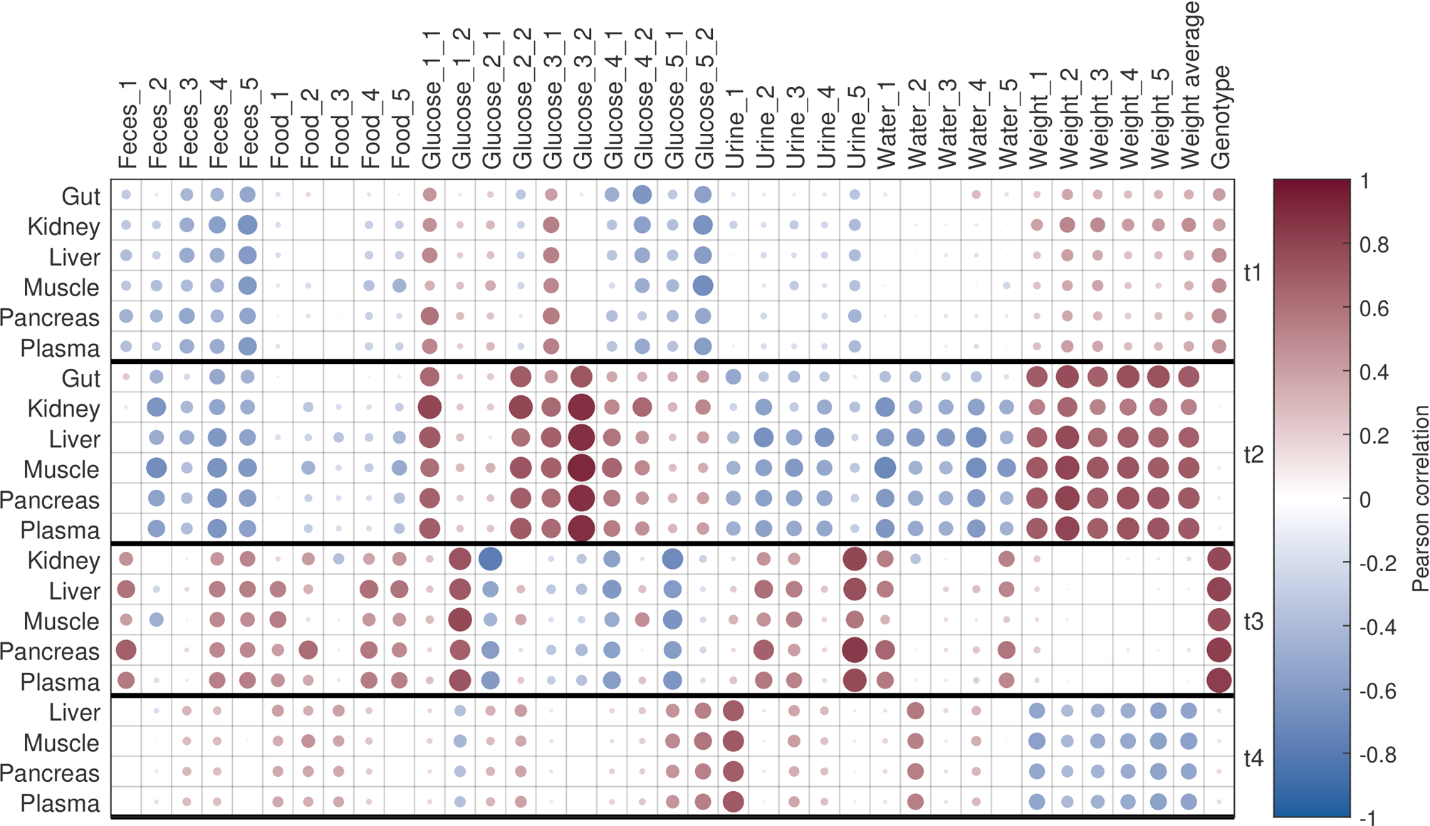


**Figure S1: Metadata correlation matrix plot.** The metadata variables (faeces, food, glucose levels, urine, water and weight, where the number represents the day of sampling i.e. _1 was measured on day 1. Blood glucose was measured both fasting and after feeding i.e. _1_1 represents fasting blood glucose on day 1 of experiments.). The correlation matrix plot shows the Pearson correlation between these metadata variables and the variation found by the JUMBA model. Globally joint t2 and t4 shows strong correlation to weight, and locally joint t3 to the genotype.


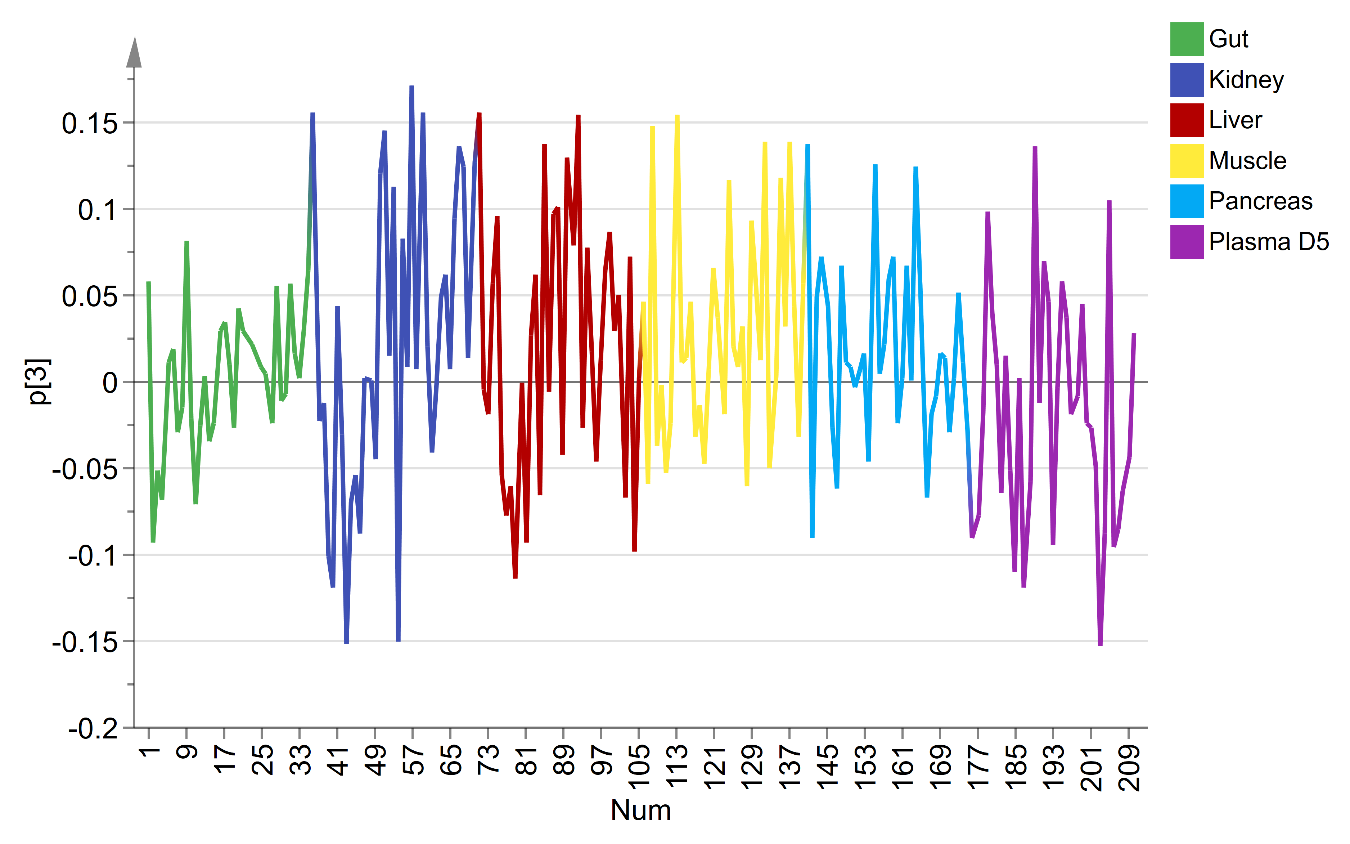


**Figure S2: Concatenated PCA loading p3**. Coloured by tissue.


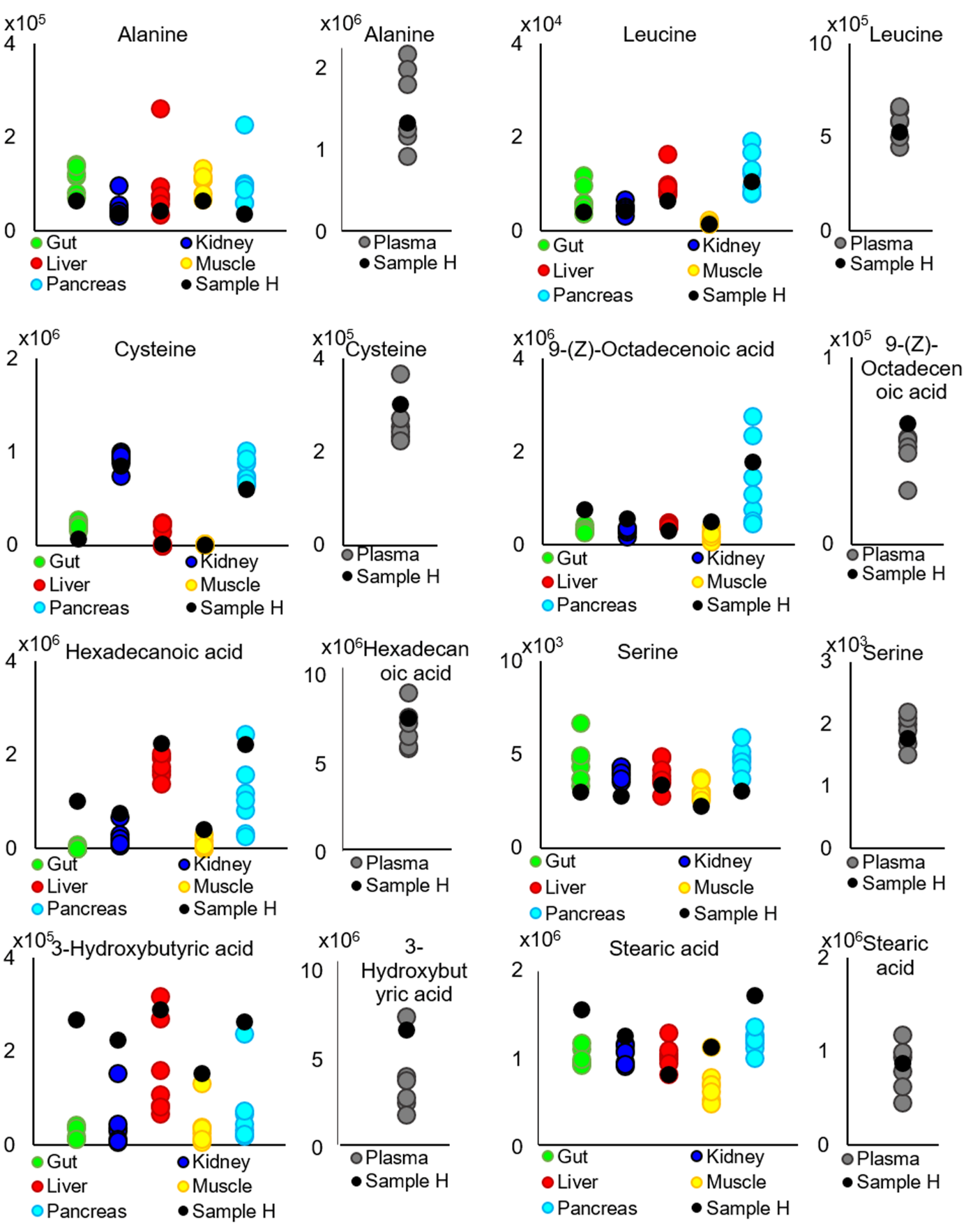


**Figure S3.** **Mouse H relative concentrations.** First globally joint JUMBA component explained the variation of mouse H. The relative concentrations of the metabolites where mouse H (marked in black) displayed the most extreme concentrations in three tissues or more.


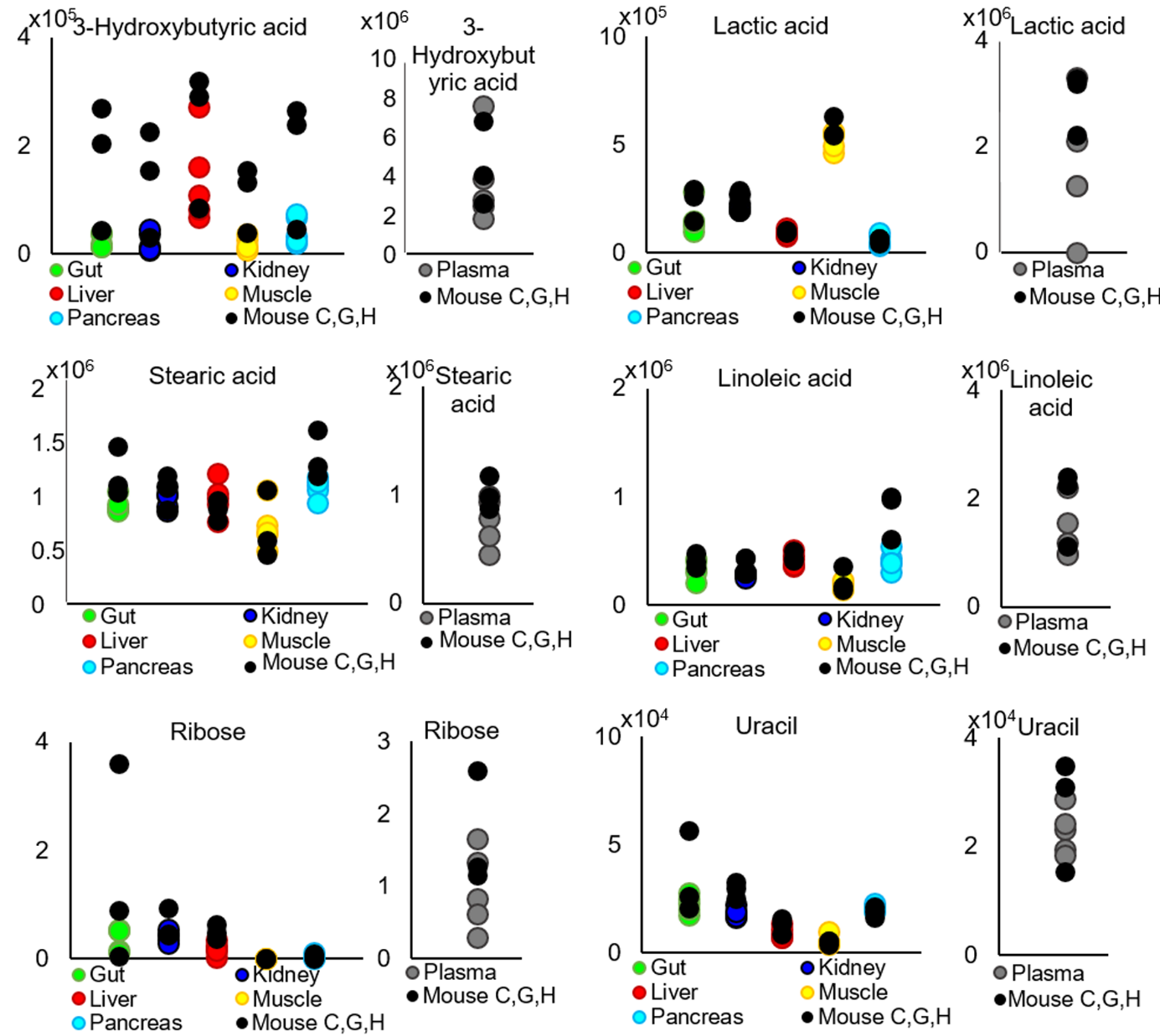


**Figure S4. Mouse C, G and H relative concentrations**. The second globally joint JUMBA component explained the separation between the three largest mice and a cluster of the five smaller mice. The black sample are the more diverse mice C, G and H.


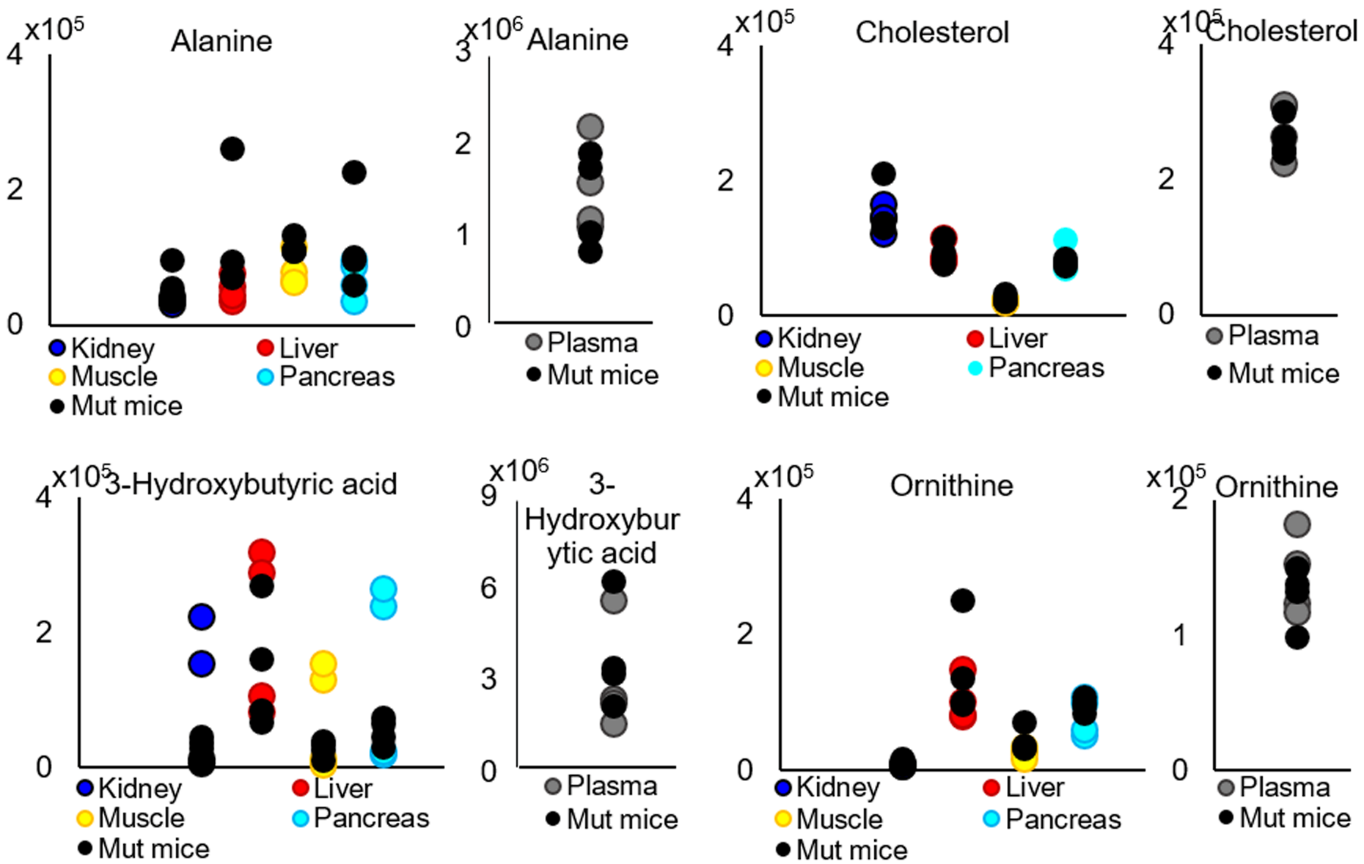


**Figure S5. MODY5/RCAD mutation vs wild type (wt).** Metabolites that were found to differ between MODY5/RCAD mutation (mouse A, B, C and D) and wt mice. Wild type mice are seen in black and wild type in colour. The plasma loadings had a negative correlation with the organ loadings (seen here as opposite directions in plasma compared to organ tissues). The gut samples not shown as they were not contributing to this component.


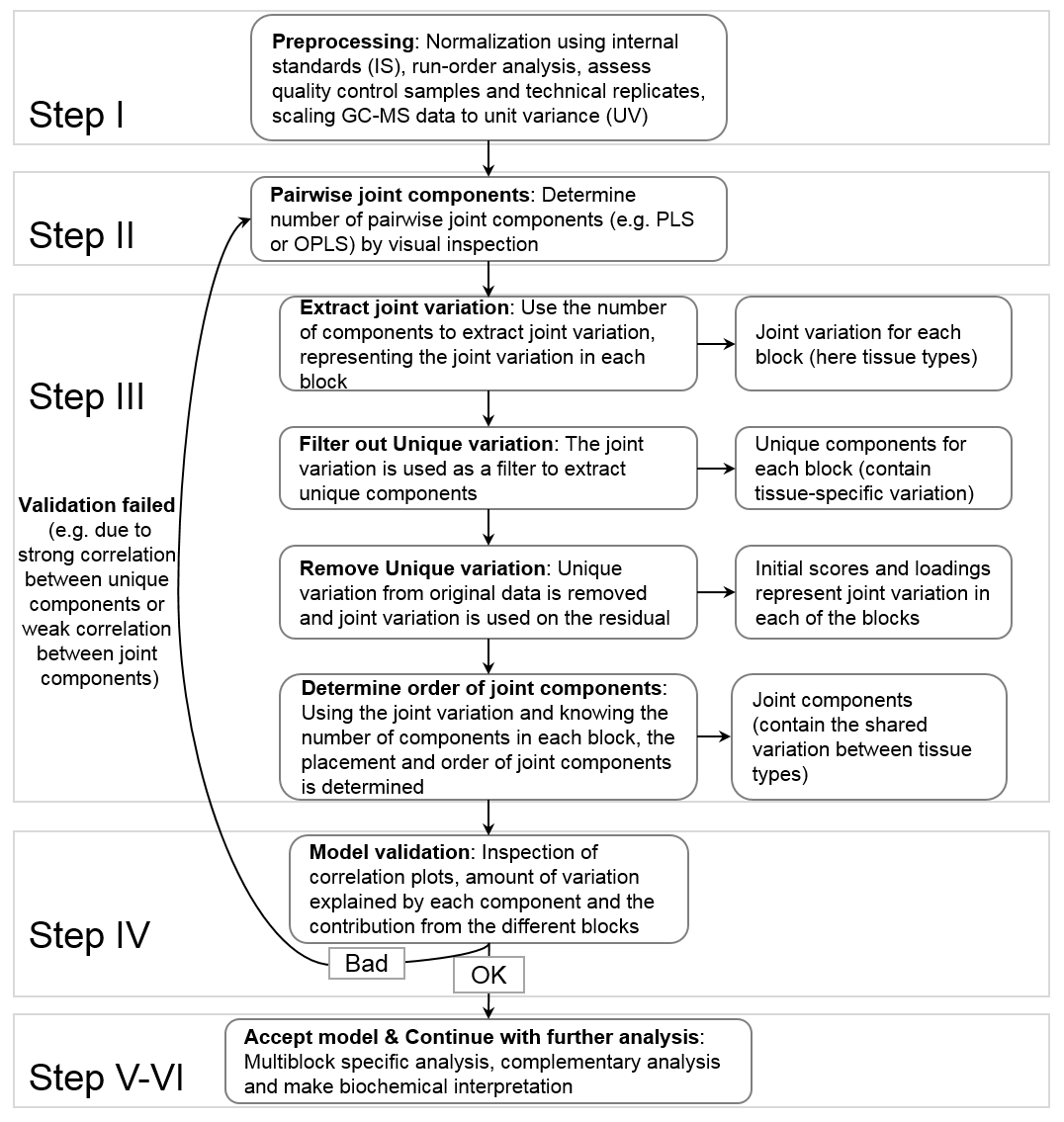


**Figure S6. JUMBA Workflow overview**. When working with small data sets the model creation is at large risk of overfitting compared to the case with many samples, where cross-validation is used to select the number of pairwise joint components. In our case the low degree of freedom forced us to select maximum one pairwise joint component. Modified from Skotare *et al.* where a similar overview is available for comparison between the case of many samples versus the case of few samples, presented here.

Skotare, T.; Nilsson, D.; Xiong, S.; Geladi, P. and Trygg, J. Joint and Unique Multiblock Analysis for Integration and Calibration Transfer of NIR Instruments. *Anal. Chem*. **2019**, *91(5),* pp. 3516-3524. doi: 10.1021/acs.analchem.8b05188.

## Materials and Methods

### S.1. Materials

All standard reagents were of analytical grade or equivalent and obtained from Sigma-Aldrich (St Louis, MO, USA), Merck (Darmstadt, Germany) and J.T. Baker (Phillipsburg, NJ, USA). N-Methyl-N-trimethylsilyltrifluoroacetamide (MSTFA) plus 1 % trimethylchlorosilane (TMCS) and pyridine were obtained from Thermo Fisher Scientific (Rockford, IL, USA). The stable isotope-labelled internal standards, [^13^C_5_]-proline, [^2^H_4_]-succinic acid, [^13^C_5_,^15^N]-glutamic acid, [1,2,3-^13^C_3_]-myristic acid, [^2^H_7_]-cholesterol and [13C4]-disodium α-ketoglutarate were purchased from Cambridge Isotope Laboratories (Andover, MA); [^13^C_6_]-glucose, [^13^C_12_]-sucrose, [^13^C_4_]-hexadecanoic acid and [^2^H_4_]-putrescine were purchased from Campro (Veenendaal, The Netherlands) and [^2^H_6_]-salicylic acid was obtained from Icon (Summit, NJ). Stock solutions of each internal standard were prepared in either Milli-Q water or methanol to a concentration of 0.5 µg/µL.

### S.2. Metabolite extraction from plasma

Frozen plasma was thawed at room temperature for 10 min and stored on ice (4 °C). Plasma samples were prepared for GC-TOF-MS analysis by adding 900 µl extraction mix (methanol:H_2_O, 8:2, v/v), containing all eleven isotopically labelled internal standards (7 ng/µl), to 100 µl aliquots of plasma. Each sample was extracted vigorously using a MM 400 Vibration Mill (Retsch GmbH & Co. KG, Haan, Germany) at a frequency of 30 Hz for 3 min, followed by centrifugation at 18 620 g for 15 min at 4 °C. A volume of 200 µl supernatant was transferred to a GC vial and evaporated to dryness in a SpeedVac concentrator (Savant Instrument, Framingdale, NY, USA).

### S.3. Derivatisation of samples

A 30 µl sample of methoxyamine (15 µg/µl) in pyridine was added to each GC vial and the resultant mixture was shaken vigorously for 10 min. Methoxymation was performed at room temperature for 16 h, followed by the addition of 30 µl MSTFA with 1% TMCS to each sample (brief vortex). Samples were left at room temperature for 1 h to allow silylation to occur, followed by the addition of 30 µl heptane (containing 15 ng/µl methyl stearate as an internal standard) and a brief vortex for 10 s. For selected samples technical replicates were also performed, where the whole extraction process was repeated (in triplicate) on the same plasma sample. For these triplicates, the mean values were used.

### S.4. GC-TOF-MS analysis

A volume of 1 µl of each derivatised sample was injected splitless by a CTC Combi Pal autosampler (CTC Analytics AG, Zwingen, Switzerland) into an Agilent 6980 GC equipped with a 10 m X 0.18 mm i.d. fused-silica capillary column chemically bonded with 0.18-um DB 5-MS stationary phase (J&W Scientific Folsom, CA). The injector temperature was set to 270 °C. Helium was used as the carrier gas at a constant flow rate of 1 mL min-1 through the column. For every analysis, the purge time was set to 60 s at a purge flow rate of 20 mL min-1 and an equilibrium time of 1 min. The column temperature was held initially at 70 °C for 2 min, then increased to 320 °C at a rate of 30 °C min^-1^, where it was held for 2 min. The column effluent was introduced into the ion source of a Pegasus III time-of-flight mass spectrometer (Leco Corp., St Joseph, MI). The ion source and transfer line temperatures were set to 200 °C and 250 °C, respectively. Ions were generated by a 70-ev electron beam at a current of 2.0 mA. Masses were acquired in the mass range 50-800 m/z at a rate of 30 spectra s^-1^. The acceleration voltage was turned on after a solvent delay of 150 s. The detector voltage was 1670 V.

### S.5. Data analysis of all samples analysed by GC/MS

Nonprocessed MS files from GC/TOF-MS analysis were exported in NetCDF format to MATLAB software 7.11 (Mathworks, Natick, MA), where all data pretreatment procedures, such as baseline correction, chromatogram alignment, time-window setting and multivariate curve resolution (MCR) [32] were performed using custom scripts. Automatic peak detection and mass spectrum deconvolution with ChromaTof software were performed using a peak width set to 2 s.

For the identification of metabolites, NIST MS Search 2.0 software was used to compare the mass spectra of all detected compounds with spectra in the NIST library 2.0, the in-house mass spectra library established by Umeå Plant Science Centre and the mass spectra library maintained by the Max Planck Institute in Golm (http://gmd.mpimp-golm.mpg.de/). A retention index comparison was performed, with a retention index deviation <+-10 (in addition to a high spectral match) resulting in a positive ID. LECO ChromaTOF software v4.32 (Leco Corp., St Joseph, MI) was also used as an additional tool for metabolite identification.

The data were normalised using all 11 internal standards (eluting over the whole chromatographic time range). To obtain accurate peak areas for the internal standards, 2 unique masses for each compound were specified and the samples were reprocessed using an in-house MATLAB 7.11 based script. A principal component analysis, using these peak areas, was calculated and the T-score value for each sample was used to normalise the resolved data by dividing the peak areas of each sample with the corresponding score value. Multivariate analysis was performed with SIMCA 16 software (Umetrics AB, Umeå, Sweden).
